# Evaluation of a commercial cardiac motion phantom for dual‐energy chest radiography

**DOI:** 10.1120/jacmp.v15i2.4508

**Published:** 2014-03-06

**Authors:** Ching‐Yi Hsieh, Gregory Gladish, Charles E. Willis

**Affiliations:** ^1^ Department of Imaging Physics The University of Texas MD Anderson Cancer Center Houston TX; ^2^ Department of Diagnostic Radiology The University of Texas MD Anderson Cancer Center Houston TX 77030 USA

**Keywords:** dual‐energy subtraction chest radiography, misregistration, cardiac motion phantom, heart rate, ejection fraction

## Abstract

Misregistration due to cardiac motion causes artifacts in two‐exposure dual‐energy subtraction images, in both the soft‐tissue‐only image and the bone‐only image. Two previous investigations have attempted to avoid misregistration artifacts by using cardiac gating of the first and second exposures. The severity of misregistration was affected by the heart rate, the time interval between the low‐ and high‐energy exposures, the total duration of the two exposures, and the phase of the cardiac cycle at the start of the exposure sequence. We sought to determine whether a commercial phantom with a simulated beating heart can be use to investigate the factors affecting misregistration in dual‐energy chest radiography. We made dual‐energy images of the phantom in postero–anterior orientation using the indirect digital radiography system (GE XQ/i). We acquired digital images at heart rates between 40 beats per minute and 120 beats per minute and transferred them to a computer, where the area of the artifact on the silhouette of the heart was measured from both soft‐tissue‐only and bone‐only images. For comparison, we measured misregistration in clinical dual‐energy subtraction images by the same method. Generally speaking, without synchronization of the exposure sequence with the cardiac cycle, the area of the misregistration artifact increased with heart rate for both the phantom and clinical images. However, the phantom exaggerated the magnitude of misregistration relative to clinical images. Although this phantom was designed for horizontal operation and computed tomography imaging, it can be use in an upright configuration to simulate heart motion for investigation of dual‐energy misregistration artifacts and control.

PACS numbers: 87.59.bf, 87.57.cf, 87.57N

## I. INTRODUCTION

**Figure 1:**
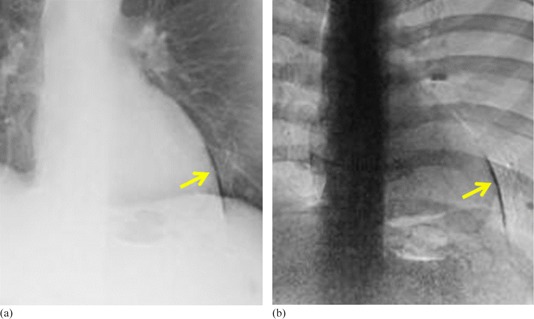
Human DES chest images showing (a) soft‐tissue‐only and (b) bone‐only subtracted images. Yellow arrows indicate misregistration of the left ventricle.

Dual‐energy subtraction (DES) X‐ray imaging offers a promising adjuvant to conventional chest radiography for accurate, early detection of lung disease. DES images reduce the influence of anatomical noise and offer the potential for analysis of nodule calcification. DES images produced from two sequential exposures are subject to artifacts in both the soft‐tissue‐only and bone‐only images because of cardiac motion, as shown in Fig. 1. The degree of misregistration is affected by the heart rate, the time interval between the low‐ and high‐kilovoltage potential (kVp) exposures, the total duration of the two exposures, and the phase of the cardiac cycle at the start of the exposure sequence.^1^, ^2^

Any object that exhibits periodic motion could be use to study temporal misregistration in DES. In fact, a simple clock phantom was used previously.^(3)^ However, this phantom was not patient‐equivalent with respect to the attenuation of X‐rays and was not anthropomorphic. For our systems, DES examinations can be performed only by using automatic exposure control (AEC). The clock phantom required additional polymethyl methacrylate to deliver clinically realistic beam current (mAs) when using AEC.

Previous investigators avoided misregistration artifacts by cardiac gating of both the first and second exposures. One use the electrocardiograph (ECG) signal,^(1)^ and the other used the output from a pulse oximeter.^(2)^ Both methods required a computer system that predicted the cardiac cycle and controlled the initiation of the second exposure. Both systems have been patented,^(45)^ but neither is available in a commercial medical imaging system. It is interesting that Sabol et al.^(1)^ used an anthropomorphic phantom to evaluate minimization of the misregistration artifact when a cardiac gating device was used, while Shkumat et al.^(2)^ assessed reduction of artifacts on clinical images. The anthropomorphic phantom used by Sabol et al. was custom‐built; our study used a commercial phantom. Both Sabol et al. and Shkumat et al. used computer programs that observed and predicted heart rates in order to synchronize exposures in real time. However, neither study reports the expected magnitude or distortion of misregistrations as a function of heart rate or ejection fraction.

Although our hospital has been performing DES routinely on patients since 2005, the study of artifacts on clinical images is complicated by uncontrolled variables, including patient size, body habitus, cardiac health, and other medical conditions, as well as human subject controls and privacy issues. Since it is not easy to perform a systematic, well‐controlled investigation of artifacts on actual patients, we reasoned that a cardiac motion phantom might provide useful information about the factors that affect the incidence and magnitude of misregistration artifacts.

In this study, we investigated a commercial phantom of the thorax that simulates a beating heart to determine its suitability for evaluating misregistration artifacts in DES. We tested whether the phantom was sufficiently patient‐equivalent with respect to AEC and morphology, and whether it could be operated in the vertical position required for upright exposure stations. We compared DES images of our phantom to clinical images to determine whether the artifacts seen with the phantom were similar to those observed in humans with respect to appearance, incidence, and magnitude. We measured the wall motion of the phantom heart and compared it to published data on the human cardiac cycle. We simulated wall motion at different heart rates, predicted misregistration, and compared phantom and human synthetic data to our experimental data.

## II. MATERIALS AND METHODS

### A. cardiac motion phantom

**Figure 2:**
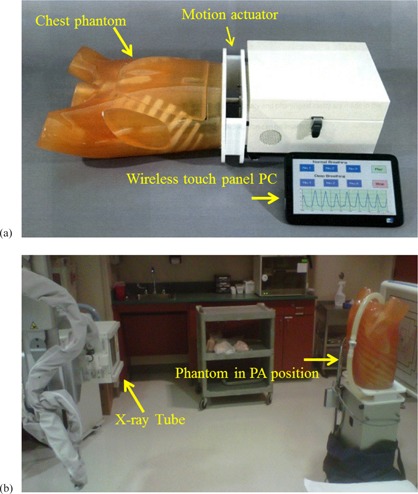
Cardiac motion phantom (a) components and (b) vertical position for image acquisition. PC = personal computer; PA = postero–anterior.

A commercial cardiac motion phantom (Model No. PH‐DCP; Fig. 2(a)) was provided for evaluation by the manufacturer (Kyoto Kagaku, Kyoto, Japan). The phantom consists of an anthropomorphic torso that includes skeletal features, a heart, and a diaphragm. Motion of the heart and diaphragm are computer controlled. The heart is filled with water and is expanded and contracted by a pump. Operator controls include respiration rate, heart rate, heart volume, and ejection fraction (EF).

### B. Image acquisition

**Figure 3:**
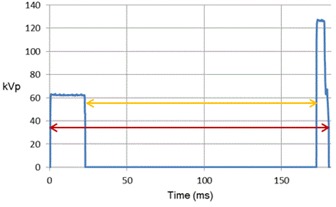
Voltage waveform of XQ/i during a single dual‐energy acquisition of CDRH LucAl phantom. First exposure was at low energy, 62 kVp for the XQ/i. Second exposure was at high energy, 125 kVp. The orange arrow represents the time interval between the end of the first exposure and the beginning of the second exposure. The red arrow represents the total exposure time. The duration of pulses is listed in Table 1.

Two‐exposure DES images of the cardiac motion phantom were acquired in postero–anterior orientation (Fig. 2(b)) with a Revolution XQ/i indirect digital radiography system (GE Healthcare, Milwaukee, WI). Consistent with local clinical practice, the images were produced at 62 kVp and 125 kVp for the XQ/i without synchronization of exposure with the cardiac cycle. The duration of each exposure appears in the Digital Imaging and Communications in Medicine (DICOM) header of the unprocessed (raw) images (GROUP, ELEMENT = 0018,1150). The time interval between low‐ and high‐kVp exposures shown in Fig. 3 was determined from the voltage waveform measured noninvasively with a RADCAL (Model 9010) dosimeter (Radcal Corp., Monrovia, CA) for each X‐ray unit during the previous annual inspection.

### C. Image analysis

**Figure 4:**
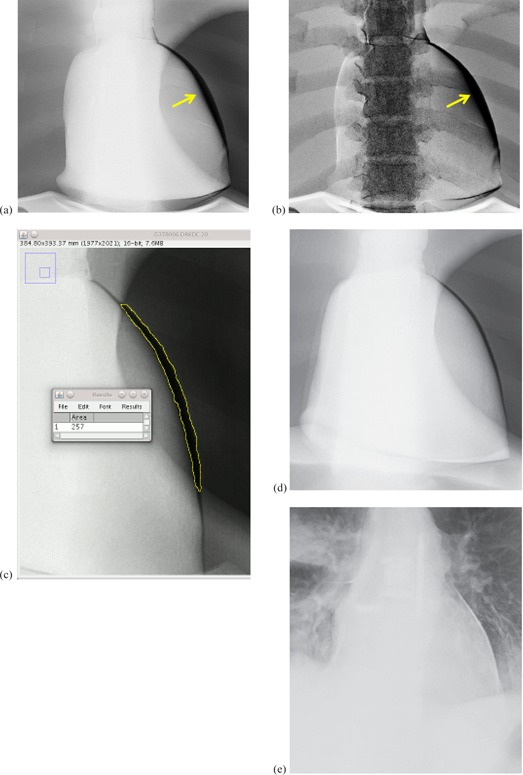
DES images showing: (a) soft‐tissue‐only and (b) bone‐only subtracted phantom images with yellow arrows indicating misregistration of the left ventricle (cf Fig. 1); (c) area of misregistration (outlined in yellow) measured by the freehand region‐of‐interest (ROI) tool on the soft‐tissue‐only image; (d) soft‐tissue‐only DES image of the phantom where misregistration is 1.9 cm^2^; and (e) soft‐tissue‐only DES image of a patient where misregistration is 0.9 cm^2^.

We transferred DICOM DES chest X‐ray images from the acquisition workstation to our Picture Archiving and Communications System (PACS; Philips iSite, Foster City, CA). The images were then exported to a personal computer, where the area of the artifact on the cardiac silhouette was measured from both soft‐tissue‐only (Fig. 4(a)) and bone‐only images (Fig. 4(b)), using the free‐hand region of interest (ROI) tool of ImageJ (National Institutes of Health, Bethesda, MD; Fig. 4(c)). For clinical images (Fig. 4(e)), the contrast in the region of the cardiac silhouette was often limited, making determination of the extent of misregistration difficult. The contrast was improved by summing the soft‐tissue‐only and the bone‐only images in ImageJ before measurement.

### D. Phantom studies

#### D.1 Pilot study

We first determined whether the phantom could be operated in a vertical orientation, whether it would cause clinically representative exposure duration under AEC, whether the phantom images would resemble human chest images, and whether the subtracted images would exhibit misregistration artifacts that were similar to those seen on clinical images.

#### D.2 Evaluation of phantom cardiac motion

**Figure 5:**
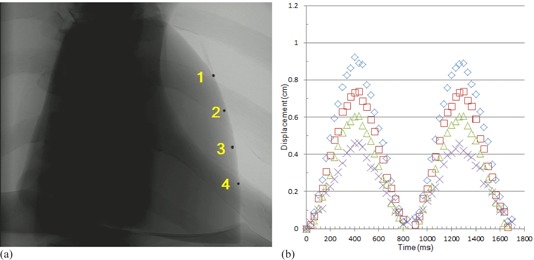
Nipple markers indicate motion of phantom cardiac silhouette: (a) position of markers (BBs) on left lateral cardiac wall; (b) displacement of markers during two cardiac cycles at 70 bpm. (◊) displacement of number 1 BB, (┚) displacement of number 2 BB, (Δ) displacement of number 3 BB, and (×) displacement of number 4 BB.

We imaged the phantom fluoroscopically to determine the time course of its motion in postero–anterior orientation. Four radioopaque nipple markers (BBs) were attached on the surface of the phantom heart, approximating the location of the left ventricle (Fig. 5(a)). Phantom operational parameters were set to a heart rate of 70 bpm and EF of 60%, with diaphragm motion disabled. We imaged the phantom using an AXIOM‐Artis angiography system (Siemens Healthcare, Malvern, PA) in acquisition mode at 30 frames/sec. DICOM images were transferred to our PACS and exported to a personal computer, as already described. We analyzed the native images by using ImageJ. The initial positions of each marker were taken from the first frame. Displacement of each marker in subsequent frames was measured with respect to its initial position.

#### D.3 Evaluation of misregistration vs. heart rate

We then evaluated the dependence of misregistration on heart rate, acquiring images at rates from 40 beats per minute (bpm) to 120 bpm with EFs of 40%, 50%, and 60% and without synchronization of the initiation of the exposure sequence with the cardiac cycle. Diaphragm motion was disabled. “Heart volume” (stroke volume) was fixed at 70 cm^3^. We imaged the phantom four times at each combination of heart rate and EF and calculated the average misregistration for each combination of heart rate and EF. We use conventional statistical methods to assess correlations between the misregistration area and heart rate, and EF.

### E. Clinical studies

#### E.1 Retrospective review

We collected and analyzed images and data from patients undergoing DES who had also undergone prior DES exams to evaluate the relationship between misregistration and heart rate. A waiver of informed consent was granted by our center's Institutional Review Board. The subject population included both men and women. The enrollment process began by checking the list of patients who had undergone a DES exam on a chosen day, and then looking for prior DES exams for each patient without regard for the presence or absence of artifacts. Any adult patient (age ≥ 18 years) who already had undergone at least two DES exams, each of which was accompanied by a resting heart rate or ECG contemporary with the exam, was selected for this study. Exams that were not associated with a documented heart rate or ECG were excluded. If a patient underwent a new exam during the course of data collection, that exam was included if accompanied by heart rate or ECG data.

Enrollment occurred on nine different days, and no more than five patients were enrolled on a single day. Because DES exams have been conducted at our institution routinely for five years, we were able to enroll 23 patients who had undergone multiple DES exams, for a total of 131 DES exams. All exams were performed between February 6, 2007 and August 22, 2012.

The process for downloading images was the same as the process for creating phantom images. The patient's resting heart rate was recorded from the date closest to the DES exam, usually on the day of the exam or within one day before or after. Only a handful of exams included in the study had an interval greater than one day between the recorded heart rate and the exam.

The area of misregistration was measured from DICOM images downloaded from our PACS, as already described. Misregistration could present at the silhouette of the heart in the vicinity of the right atrium, the right ventricle, the left atrial appendage, and the left ventricle. Misregistrations also occur in the region of the aortic arch, but these were not measured in this study. Misregistration at the right ventricle was difficult to assess because of the proximity to the diaphragm and was excluded. Misregistrations at the left ventricle were on average twice as large as those at the right atrium and three times those at the left atrial appendage. For these reasons, further analysis was restricted to left ventricular (LV) misregistration.

The retrospective study protocol did not include recording the patient's EF. Because our patient population comprises cancer patients rather than cardiac patients, we assumed that their cardiac function varies about the same as in the general population with the same age range. The study population was not controlled for age, sex, medical condition, or reason for exam.

#### E.2 Review of image quality complaints

In addition to the 131 exams chosen for the retrospective review, a number of other exams had been identified by radiologists in the course of routine clinical operations as having poor image quality secondary to misregistration artifacts. From this group, five patients who underwent a total of 27 DES exams were selected. Although misregistration was severe enough to trigger a radiologist complaint in one of each of these patients' exams, misregistration was evident in other exams for the same patient, sometimes greater than in the exam that generated the complaint. A two‐tailed *t*‐test comparing misregistrations measured from these images to those measured from the retrospective review indicated no statistically significant difference between the two distributions (p=0.83), so these data were pooled for further analysis.

The total patient population included 17 men and 11 women, ranging in age from 36 to 82 years, with an average age of 62 years. Of the clinical DES exams, 158 were performed on an XQ/i system.

### F. Simulation

On the basis of the results of the phantom cardiac motion study, we were able to simulate the distribution of misregistration artifacts that would be expected with a fixed delay between the low‐kVp and high‐kVp exposures at a variety of phantom heart rates. Using representative data from the literature^(6)^ describing the time course of human left ventricular volume and approximating the volume as an ellipsoid, we were able to make a simplistic model of wall displacement over the cardiac cycle and to calculate a distribution of misregistration artifacts expected in clinical exams for comparison to those produced by the phantom.

## III. RESULTS

### A. Pilot study

**Figure 6:**
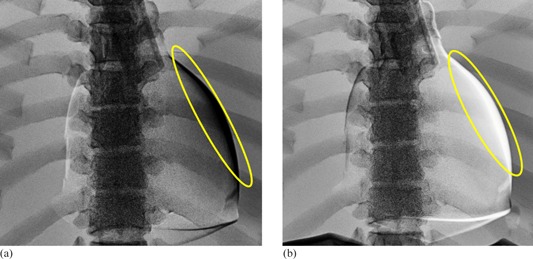
Misregistration in two bone‐only subtracted images of the phantom: (a) artifact (within yellow line) appears dark; (b) artifact appears light (see Results section B for explanation).

Table 1 shows the low‐ and high‐energy exposure times, the time interval between the end of the first exposure and the beginning of the second, and the total time of the exposure sequence. For comparison, the duration of AEC exposures during annual testing using the LucAl chest phantom (Center for Devices and Radiological Health, Silver Spring, MD)^(7)^ is shown for each machine. The LucAl chest phantom is designed to rpresent an adult of average dimensions (22.5 cm thick thorax). We estimated the size of the patient that the phantom represents on the basis of the work of Shah et al.^(8)^ Using the mAs delivered at 125 kVp with the LucAl phantom (2.8 mAs) and the mAs delivered for the cardiac motion phantom (1.6 mAs), and assuming a half‐value layer for the thorax of 9.6 cm, the thickness of the thorax corresponding to the cardiac motion phantom is only about 15 cm. This thickness is more like that of a child than an adult; therefore, the cardiac motion phantom behaves more like an adult of smaller‐than‐average dimensions. The time interval between the end of the first exposure and the beginning of the second exposure was relatively constant (±0.4%). DES images of the phantom (Fig. 4) showed misregistration artifacts similar to those observed in clinical images (Fig. 1), including both light and dark artifacts (Fig. 6).

**Table 1:** Precision of DES acquisition

*Phantom*	*Low‐Energy Exposure Time (ms)*	*Time‐Internal (ms)*	*High‐Energy Exposure Time (ms)*	*Total Acquisition Time (ms)*
Cardiac motion phantom	8.0±2.3	149.2±0.6	2.5±0.6	159.7±2.5
CDRH LucAl chest phantom	18.0	150.7	4.0	172.7

CDRH=Center for Devices and Radiological Health

### B. Evaluation of phantom cardiac motion

Figure 5 shows the location of four nipple markers on the left lateral aspect of the phantom heart and their displacement during the phantom cardiac cycle. All four markers exhibited sinusoidal periodic motion (Fig. 5(b)). The magnitude of displacement decreased gradually from the superior to the inferior marker; however, the period of their motion appears identical (0.86 sec) and agrees with the phantom heart rate control setting of 70 bpm. The initial frame of the fluoroscopic acquisition fortunately coincided with the beginning of the phantom's cardiac cycle.

**Figure 7:**
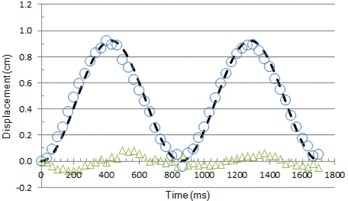
Displacement of marker as sinusoidal function. (○) displacement of superior marker (number 1 BB); (—) displacement calculated from Eq. (1); (Δ) difference between measured and calculated displacement.

The motion of the superior marker, shown in Fig. 7, can be represented adequately by Eq. (1):

(1) x=0.46[1−cos(1.17×2πt)]

where × is the displacement in cm, *t* is the time in sec, 1.17 is the frequency in beats per second, and 0.46 is one‐half the maximum displacement in cm. Figure 7 shows the wall motion comparison between the first marker movement and Eq. (1). The largest difference between the first BB and Eq. (1) in Fig. 7 was less than 0.1 cm. The average displacement difference was −0.02±0.39 cm. Similar equations describe the motion of the remaining markers, substituting appropriate values for one‐half maximum displacement.

**Figure 8:**
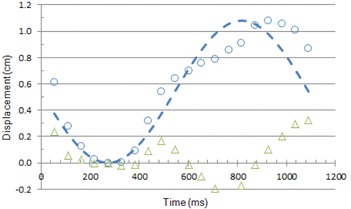
Displacement of the left ventricular wall of the human heart compared to that of the phantom. (○) displacement of human heart calculated from left ventricular volume; (—) displacement of phantom calculated from Eq. (1) scaled for 55 bpm and 1.08 cm maximum displacement; (Δ) difference between human heart and phantom displacement.

For comparison, the displacement of the left ventricular wall of a human heart was calculated from left ventricular volume data reported in the literature.^(8)^ Equation (1) was scaled to match the human data for heart rate (55 bpm) and maximum displacement (1.08 cm) and was phase‐shifted (π/2) to synchronize the minima of the two functions. A comparison of human left ventricular motion and phantom motion is shown in Fig. 8. The maximum difference in displacement was 0.33 cm. The average difference in displacement was 0.12±0.10 cm. Within these limits, the left ventricular wall motion of the phantom was similar to that of a human heart.

### C. Evaluation of misregistration vs. heart rate

The aggregate results of phantom misregistration, summarized in Table 2, include the maximum, average, and standard deviation (SD). Figure 4(d) shows one of the soft‐tissue‐only DES images of the phantom. Table 3 reports the average misregistration and SD for all heart rates and EFs for the XQ/i. Considering the large SDs of these measurements in comparison to the means, the relationship is not evident from the tabular data alone.

**Figure 9:**
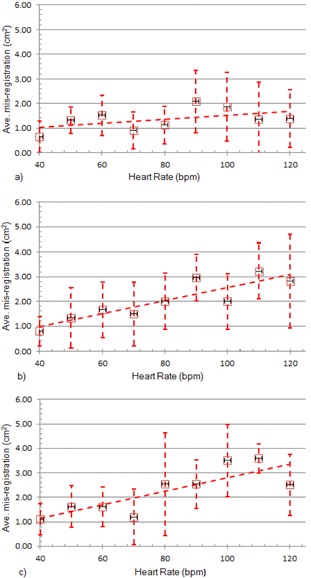
Phantom misregistration as a function of heart rate for: (a) $\text{EF}=40\%,r^{2}=0.26$; (b) $\text{EF}=50\%,r^{2}=0.79$; and (c) $\text{EF}=60\%,r^{2}=0.68$. (◻)(—) XQ/i. Error bars indicate ±1 SD.

Figure 9 shows the data from Table 3 in graphical form. The trend toward larger misregistration with higher heart rate is more apparent from the regression lines and all three EFs, with the exception at 40% EF. The regressions were obtained from a linear least squares fit.

**Figure 10:**
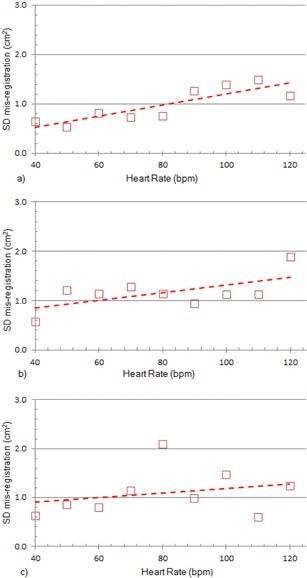
Variation in phantom misregistration as a function of heart rate for: (a) $\text{EF}=40\%,r^{2}=0.74$; (b) $\text{EF}=50\%,r^{2}=0.52$; and (c) $\text{EF}=60\%,r^{2}=0.12$. (◻)(—) XQ/i.

Figure 10 shows the relationship between the variation of misregistration with heart rate. The trend toward greater SD with higher heart rate is apparent from the regression lines and for all EFs, except at 40% EF.

**Table 2:** Summary of cardiac motion phantom misregistration

*N*	*Maximum (cm^2^)*	*Average (cm^2^)*	*SD*
108	5.0	1.9	1.3

N=number; SD=standard deviation.

**Table 3:** Phantom misregistration as a function of heart rate and ejection fraction

	*Misregistration (cm^2^)*		
*Heart Rate (bpm)*	*EF 40%*	*EF 50%*	*EF 60%*
40	0.7±0.6	0.8±0.6	1.1±0.6
50	1.3±0.5	1.3±1.2	1.6±0.9
60	1.5±0.8	1.7±1.1	1.6±0.8
70	0.9±0.7	1.5±1.3	1.2±1.1
80	1.1±0.8	2.0±1.1	2.4±1.9
90	2.1±1.3	3.0±0.9	2.5±1.0
100	1.9±1.4	2.0±1.1	3.5±1.5
110	1.4±1.5	3.2±1.1	3.6±0.6
120	1.4±1.2	2.8±1.9	2.5±1.2

bpm=beats per minute; EF=ejection fraction.

These results indicate that the heart rate and EF are two variables affecting misregistration. to reveal the extent to which each variable contributes to misregistration, two‐way analysis of variance (ANOVA) was applied to the data from Table 3.

Table 4 reports the results of ANOVA. The heart rate was a highly significant factor in misregistration. The EF was a highly significant factor, as well. No significant covariance is apparent.

Multiple regression analysis was also performed on the misregistration data from Table 3. The results are shown in Table 5. In this dataset, misregistration had a highly significant linear relationship with heart rate. For this dataset, there was a highly significant linear relationship between misregistration and EF. However, the magnitude of the dependence of misregistration on EF suggests that EF may be a confounding uncontrolled variable in our clinical dataset.

**Table 4:** Two‐way ANOVA of misregistration

	*p‐value*		
*X‐ray Unit*	*Factor 1: Heart Rate (bpm)*	*Factor 2: EF*	*Factor 1 vs. Factor 2*
XQ/i	$6.8\times 10^{-4}$	$3.7\times 10^{-3}$	0.84

ANOVA=analysis of variance; EF=ejection fraction.

**Table 5:** Multiple regression analysis of misregistration

	*Factor 1: Heart Rate (bpm)*			*Factor 2: EF*		
*X‐ray Unit*	*Slope (cm^2^/bpm)*	$R^{2}$	*p‐value*	*Slope (cm^2^/EF %)*	$R^{2}$	*p‐value*
XQ/i	$2.0\times 10^{-2}$	0.68	$2.0\times 10^{-5}$	$4.4\times 10^{-2}$	0.32	$1.0\times 10^{-3}$

EF=ejection fraction; bpm=beats per minute.

### D. Retrospective review

**Figure 11:**
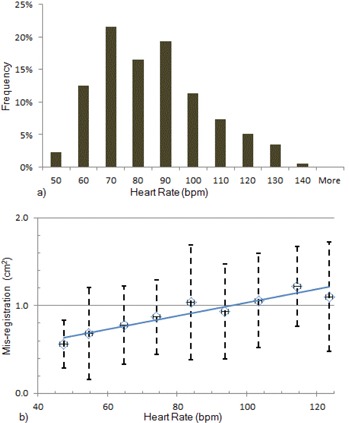
Misregistration as a function of heart rate for clinical images: (a) distribution of heart rates for all clinical exams; (b) average left ventricular (LV) misregistration versus average heart rate from clinical images stratified according to the histogram in (a). $r^{2}=0.93$; error bars indicate ±1 SD.

The distribution of heart rates for all patients and all exams is shown in Fig. 11(a). Heart rates for all patients ranged from 47 to 132 bpm, with an overall average of 81±19 bpm. The mode was 66 bpm and the median was 80 bpm. For any individual patient, the heart rate was not necessarily the same for all of that patient's DES examinations. The SD of each individual patient's heart rate was between 5 bpm and 20 bpm. Because of the wide variation of individual heart rates, misregistration data were pooled among all patients and sorted by heart rate. The maximum misregistration for all patients was 2.8 cm^2^. The average of misregistration for all patients was $0.9\pm 0.5\;{\text{cm}}^{2}$. Figure 4(e) shows one of the soft‐tissue‐only DES images of a patient. Heart rates and misregistration were averaged within intervals of 10 bpm. As shown in Fig. 11(b), the average misregistration appears to be dependent on the average heart rate; however, the uncertainty of misregistration at each average heart rate is large as well.

### E. Simulation

**Figure 12:**
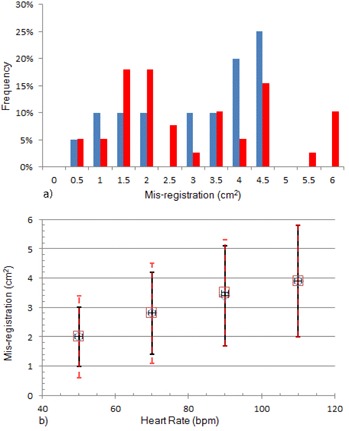
Misregistration as a function of heart rate in the simulations. Distribution of misregistrations (a) for the cardiac motion phantom (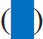) and a human heart (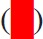) at 70 bpm. Note different maxima of the two distributions, but similar averages and SDs. Average left ventricular misregistration (b) vs. heart rate for the cardiac motion phantom (◊), $r^{2}=0.99$, and a human heart (□), $r^{2}=0.98$. Error bars indicate ±1 SD, (—) cardiac motion phantom, and (—) human heart.

Distributions of expected misregistration were simulated for the cardiac motion phantom and the human heart at four different heart rates. Values for maximum, average, and SD of misregistration are shown in Table 6, along with the most likely size of misregistration (the mode) and the likelihood of no misregistration, define as misregistration less than 0.5 cm^2^. At each heart rate, a two‐tailed Student's *t*‐test was performed to determine whether the distributions predicted for the phantom differed from those predicted for the human heart. No statistically significant difference was found. The data from Table 6 are shown graphically in Fig. 12(b). The results of the simulations indicate that the maximum and average misregistration increase with heart rate. The uncertainty of misregistration also increases with heart rate. Note the similarity between 1, 9, 12.

**Table 6:** Misregistration simulated for cardiac motion phantom and human heart

*Simulation*	*Heart Rate (bpm)*	*Maximum Mis‐R. (cm^2^)*	*Average Mis‐R. (cm^2^)*	*SD*	*Mode (cm^2^) (range (%))*	*Prob. of No Mis‐R. (%)*	*Student's t‐Test p‐value*
Phantom	50	3.2	2.0	1.0	3.0−3.5 (25%)	10	0.82
Human heart		4.9	2.0	1.3	0.5−2.0 (23%)	10	
Phantom	70	4.4	2.8	1.4	4.0−4.5 (25%)	5	0.96
Human heart		6.0	2.7	1.7	1.0−2.0 (36%)	5	
Phantom	90	5.3	3.4	1.7	5.0−5.5 (25%)	5	0.90
Human heart		6.8	3.3	1.8	2.0−2.5 (20%)	0	
Phantom	110	6.2	4.0	1.9	5.5−6.5 (30%)	4	0.95
Human heart		7.1	3.8	1.9	2.5−3.0 (15%)	0	

bpm=beats per minute; Mis‐R.=misregistration; SD=standard deviation; Prob.=probability.

## IV. DISCUSSION

### A. Sensitivity of free‐hand ROI measurement

Misregistration less than 0.5 cm^2^ cannot be distinguished easily, especially in soft‐tissue‐only images. These cases may be considered as exhibiting no misregistration. Considering a rectangular model and assuming the length of the left ventricle to be 6.9 cm, misregistration of 0. 5 cm^2^ corresponds to wall displacement of only 0.7 mm. This means that the projection of the wall moved less than 4 pixels away from its initial position.

### B. Gray level of misregistration

The misregistration of DES images could appear either black or white on the left ventricle silhouette, as seen in Figs. 6(a) and 6(b). This depends on the cardiac phase at the first and second exposures of dual‐energy acquisition. The wall position in raw images at low kVp and high kVp determines the gray level of the artifact in both the subtracted soft‐tissue‐only and bone‐only images. Dark misregistration indicates that the left ventricle was expanded at the low‐energy exposure, but was contracted at the high‐energy exposure. Conversely, light misregistration indicates that the left ventricle was contracted at the low‐energy exposure, but was expanded at the high‐energy exposure.

### C. uncertainty in clinical data

The retrospective review did not collect each patient's EF, so EF is an uncontrolled variable. Multiple regression analysis of the cardiac phantom data pooled from both X‐ray units indicated that EF is a highly significant factor on misregistration, with a strong, linear dependence on heart rate. In general, healthy individuals have an EF between 50% and 65%.^(9)^ If we assume that the EF in our patient population (average heart rate 80 bpm) varied 15%, then that introduces an uncertainty in our misregistration data on the order of $\pm 0.7\;{\text{cm}}^{2}$.

### D. Effect of magnification factor on wall motion

The apparent motion of the cardiac wall in the X‐ray units was increased by a magnification factor of 1.07 (source‐to‐image distance (SID) = 183 cm; source‐to‐object distance (SOD) = 172 cm). The apparent motion of the cardiac wall in the fluoroscopy unit was increased by a magnification factor of 1.10 (SID = 120 cm; SOD ~ 109 cm). Thus, the difference of cardiac wall motion in fluoroscopy images and the X‐ray images should be on the order of 3%. Although the geometry of projection is different in the fluoroscopic image from the DES image, the apparent effect on wall motion is small enough to be negligible.

### E. Misregistration comparisons among phantom data, phantom simulation, clinical data, and human heart simulation

As shown in Table 6, the maximum misregistration calculated from the simulation of human heart motion is larger than that for the phantom (15% to 53%). This can be appreciated from the histograms of the simulated misregistrations, shown in Fig. 12(a) for 70 bpm, even though the average and SD are similar for both distributions. This is a reflection of the difference in the rate of left ventricular wall displacement that can be seen by comparing Fig. 8 for the human heart with Fig. 7 for the phantom.

The maximum, average, and SD of misregistration in phantom images (Table 2) are smaller by approximately 30% than those of the phantom simulation data in Table 6 (6.2 cm^2^, 3.0 cm^2^, and 1.7 cm^2^, respectively). The shape of the misregistration region of the cardiac phantom is similar to half of an ellipse (Figs. 4(a) and 4(b)), but the area of misregistration in the simulations was calculated as the area of a rectangle. The simplifying assumption in the simulation overestimated the area of misregistration and may be responsible for the 30% discrepancy.

The maximum, average, and SD of misregistration for the clinical images (see Results section D above) are smaller than those of the phantom images (Table 2) by a factor of two. The maximum, average, and SD of human simulation data (Table 6) are larger than those of the clinical images, also by a factor of about two.

In our simulation of human cardiac wall motion, we use volume to determine wall displacement and assumed incompressibility of the wall. This is a gross oversimplification. Variation in wall thickness during the cardiac cycle may have contributed to the discrepancy between the simulation and clinical data.^(10)^ It also may be the case that the material comprising the phantom heart wall undergoes less variation in thickness during contraction than a human heart, so that the wall displacement is exaggerated by the phantom.

Greater wall displacement in the phantom could be caused by inaccuracy in the stroke volume in the phantom heart. If the actual stroke volume was greater than the 70 cm^3^ set by the controller, then the wall displacement would be greater than expected.

The magnitude and frequency of misregistration observed with the phantom did not always coincide with the magnitude and frequency of misregistrations observed in our clinical study. The data in Table 3 suggest that operating the phantom at a heart rate of 80 bpm and an EF of 40% would better simulate the results observed in our clinical study. The maximum misregistration for the XQ/i for all heart rates at EF of 40% was 3.7 cm^2^, which is closer to the misregistration observed in the clinical images (2.8 cm^2^). This correspondence does not imply a heart rate of 80 bpm or an EF of 40% among our cohort of patients. Considering the variability in the incidence of misregistration when exposures are not synchronized with the cardiac cycle, this may be coincidental; however, our simulations, especially as shown in Fig. 12(a), lead us to believe that repeated studies with the phantom will yield similar statistics to what we have reported.

## V. CONCLUSIONS

The cardiac motion phantom is sufficiently realistic for the study of misregistration in DES imaging using clinical X‐ray systems. The morphology of the phantom and its radiographic appearance are similar to those of a human. The attenuation properties of the phantom are high enough to operate in AEC mode; however, the quantity of mAs delivered is closer to that typically delivered for a child than to that delivered for an adult.

The phantom can be operated in vertical orientation. The periodic motion of the phantom heart differs from the pulsatile motion of the human heart; however, its misregistration artifacts are similar in appearance. The cardiac phantom exaggerates the magnitude of left ventricular misregistration artifacts observed in clinical studies. This can be minimized by operating the phantom at a low EF setting.

The maximum, average, and SD of the area of left ventricular misregistration increase with heart rate and EF. This trend was observed in phantom images, clinical images, and simulations of the phantom and human heart. If two‐image DES is performed without synchronization of acquisition with the phase of the cardiac cycle or without deformation of the original images to align clinical landmarks to compensate for cardiac motion, misregistration artifacts of this scale and variability will be present.

## ACKNOWLEDGMENTS

The authors would like to acknowledge Kyoto Kagaku Co., Ltd, for the loan of the dynamic chest and heart phantom and for instructing us on its configuration and operation. The authors appreciate constructive discussions about this project with Professors Tinsu Pan and Osama Mawlawi.
